# Supplementary material for: Co-targeting KRAS G12C and EGFR reduces both mutant and wild-type RAS-GTP
Source: NPJ Precis Oncol. 2022 Nov 23;6:86. doi: 10.1038/s41698-022-00329-w (PMC9684405; doi:10.1038/s41698-022-00329-w)
Supplement: Supplementary file 1 — Supplemental Figures [file 41698_2022_329_MOESM1_ESM.pdf]

**Supplementary Information**

# Co-targeting KRAS G12C and EGFR reduces both mutant and wild-type RAS-GTP

**Thomas McFall, Michael Trogdon, Anita C. Guizar, John F. Langenheim, Laura Sisk-Hackworth, Edward C. Stites**

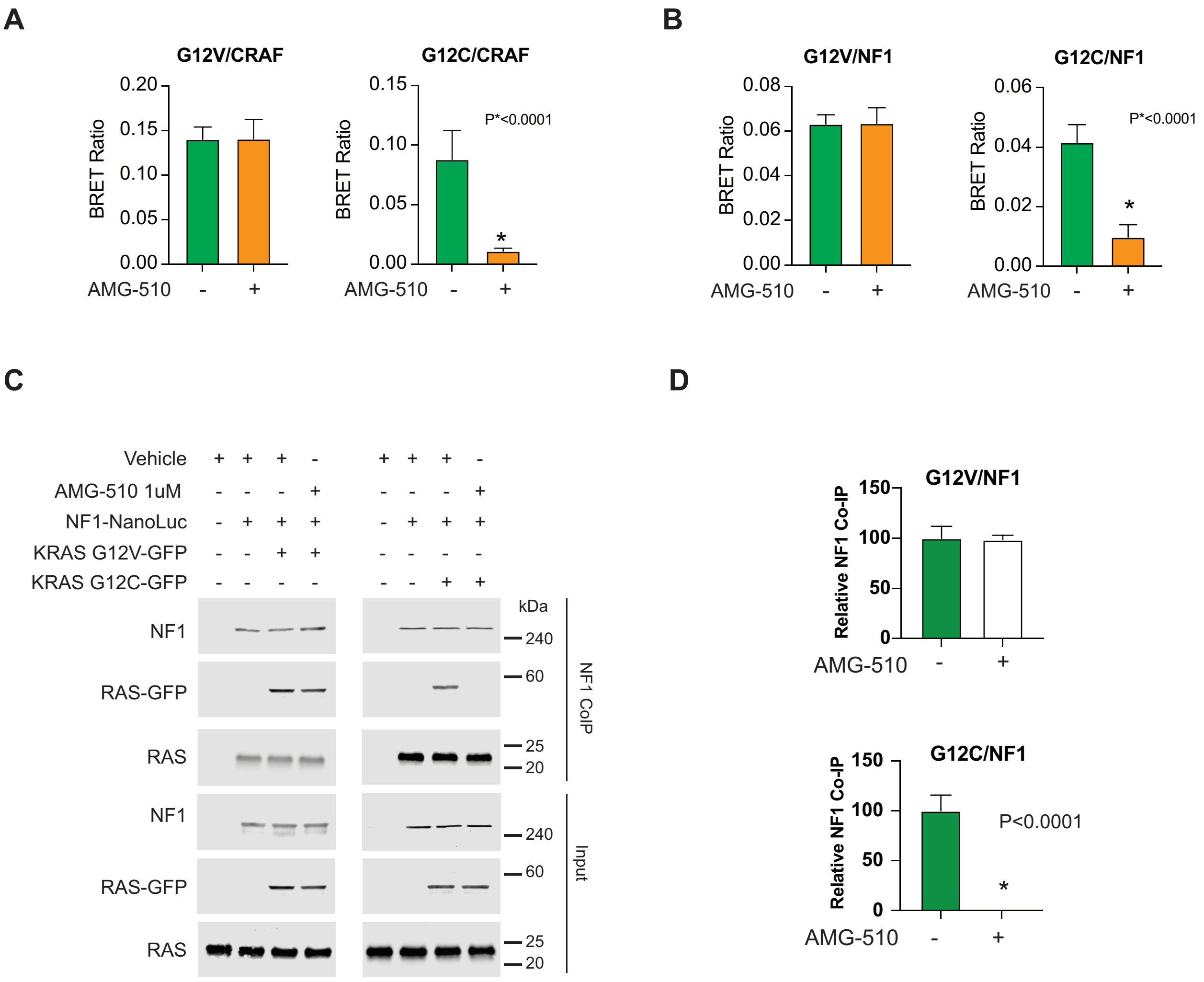


**Supplementary Figure 1. Experimental analysis of the effects of AMG-510 bound KRAS-G12C cancer cells.** (**A**) HEK293T cells were co-transfected with mutant KRAS-GFP and NF1-NanoLuc with and without 500nM AMG-510 for 24 hours and signal is represented as a BRET ratio for both sample groups. Bars represent the mean of eight biological replicates. Results are representative of an individual experiment from three separate experiments. (**B**) HEK293T cells were co-transfected with mutant KRAS-GFP and CRAF-NanoLuc with and without 500nM AMG-510 for 24 hours and signal is represented as a BRET ratio for both sample groups. Bars represent the mean of eight biological replicates. Results are representative of an individual experiment from three separate experiments. (**C**). HEK293T cells were transfected with either mutant KRAS-GFP and NF1, NF1 alone, or mock transfection. Cells were then treated with either vehicle or 500 nM AMG-510 for 24 hours. Following treatment, cells were lysed and NF1 co-IP was performed. IP product and input lysates were resolved and subjected to western blot analysis. Results are representative of an individual experiment from three separate experiments. (**D**) Mean KRAS G12C pull-down for the three independent NF1-coIP experiments. Statistical analyses were performed with unpaired t-test and P-values are indicated. Error bars in all panels represent standard deviation.

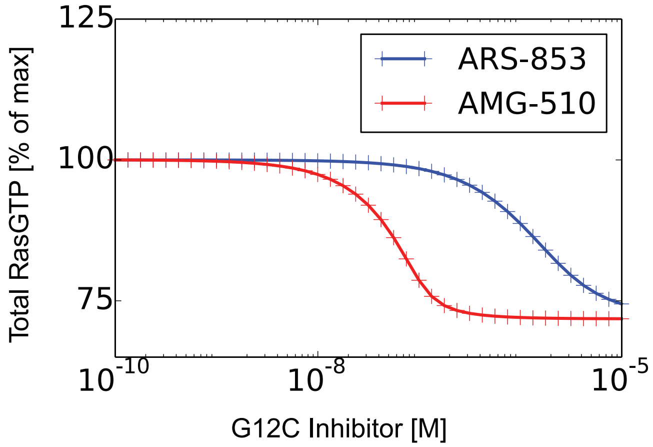


**Supplementary Figure 2. KRAS G12C inhibition mathematical model predictions for different KRAS G12C inhibitors.** Simulated G12C inhibitor dose responses from the computational RAS model for KRAS G12C for AMG-510 and for ARS-853.

# Raw images WB  Figure 1C SW837 Cells

## KRAS RBD

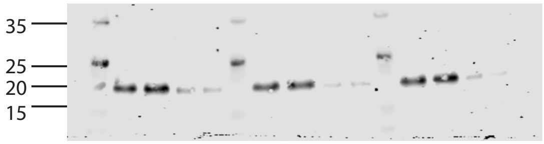


## KRAS Total

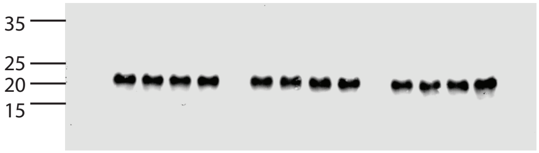


## HRAS RBD

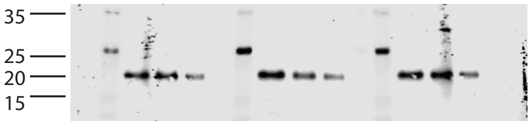


## HRAS Total

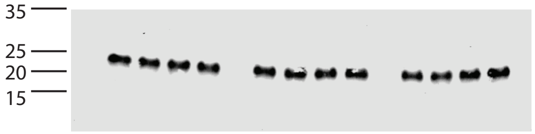


## NRAS RBD

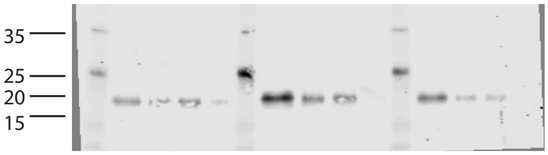


## NRAS total

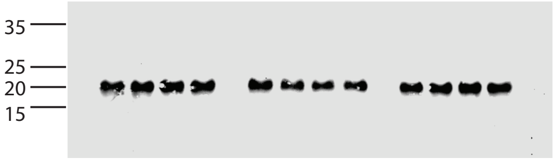


## pERK 800 nm

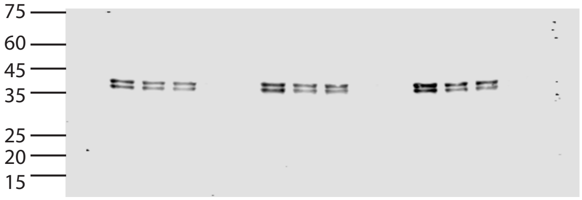


## total ERK 700nm

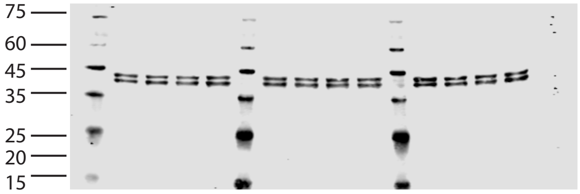


## GAPDH

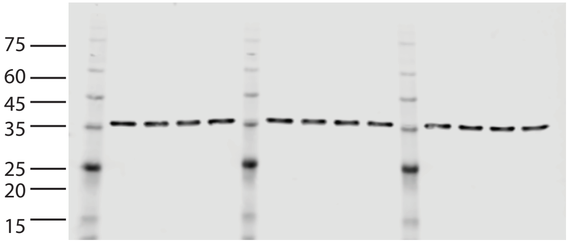


Protein lysates were run on gels to separate proteins by molecular weight.
Blots were transferred to PVDF membranes and probed with the antibodies indicated.
Membranes were imaged utilizing Licor Odyssey imager.
Each stain includes a range of molecular weight ladder of at least four markers.
Some membranes were cut to probe for multiple targets or to redcue background from RBD.
Some antibodies recognize ladders stronger than others, and intensity differences of the ladder compared to the target protein is observed.

Supplementary Figure 3

# Raw image files for Figure 1C SW1463 Cells

KRAS RBD

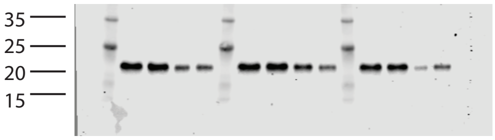


KRAS Total

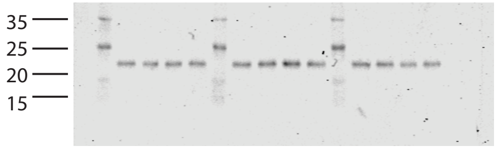


HRAS RBD

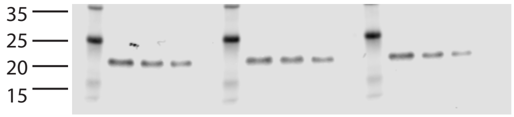


HRAS Total

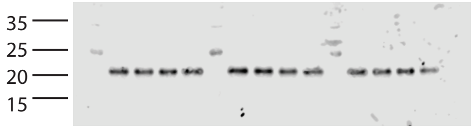


pERK

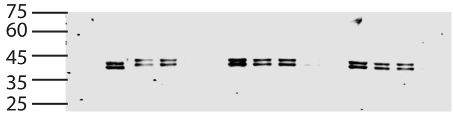


total ERK

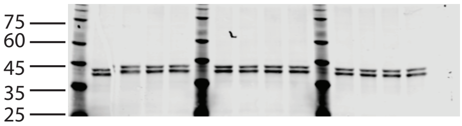


GAPDH

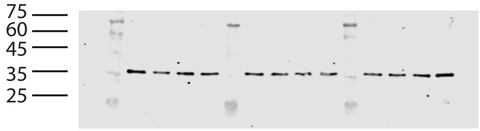


NRAS RBD

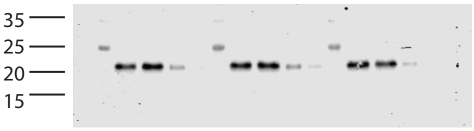


NRAS total

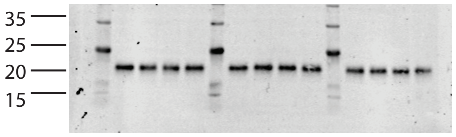


Protein lysates were run on gels to separate proteins by molecular weight.
Blots were transferred to PVDF membranes and probed with the antibodies indicated.
Membranes were imaged utilizing Licor Odyssey imager.
Each stain includes a range of molecular weight ladder of at least four markers.
Some membranes were cut to probe for multiple targets or to reduce background from RBD.
Some antibodies recognize ladders stronger than others, and intensity differences of the ladder compared to the target protein is observed.

Supplementary Figure 4

# Raw image files for Figure 1C SW403 Cells

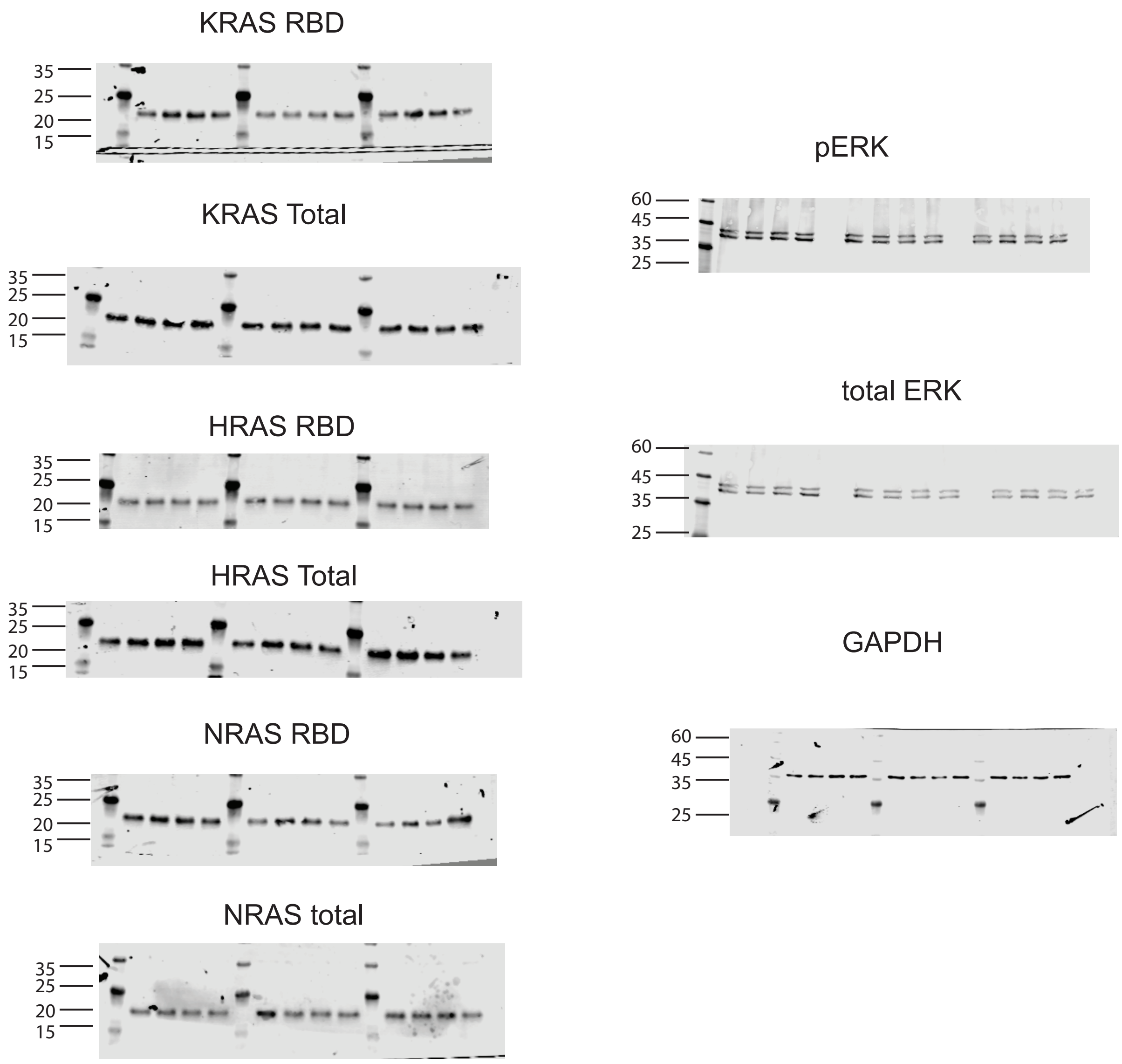


Protein lysates were run on gels to separate proteins by molecular weight.
Blots were transferred to PVDF membranes and probed with the antibodies indicated.
Membranes were imaged utilizing Licor Odyssey imager.
Each stain includes a range of molecular weight ladder of at least four markers.
Some membranes were cut to probe for multiple targets or to reduce background from RBD.
Some antibodies recognize ladders stronger than others, and intensity differences of the ladder compared to the target protein is observed.

Supplementary Figure 5

# Raw image files for Figure 1C CACO2 Cells

KRAS RBD

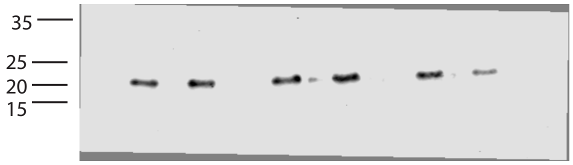


KRAS Total

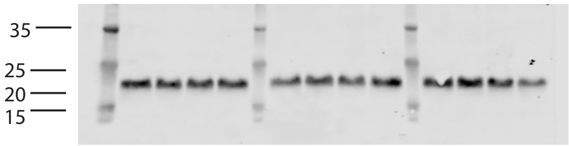


HRAS RBD

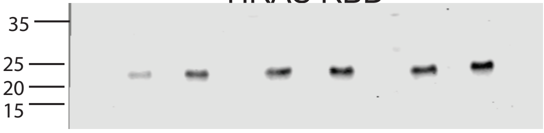


HRAS Total

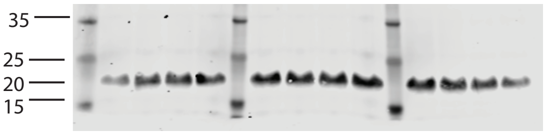


NRAS RBD

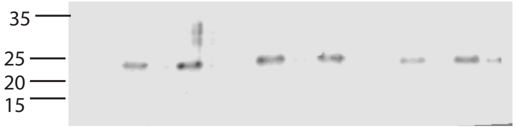


NRAS total

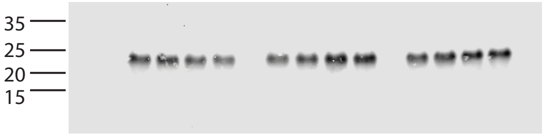


pERK

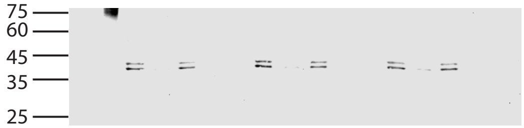


total ERK

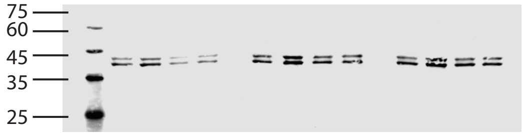


GAPDH

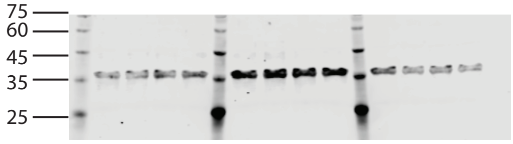


Protein lysates were run on gels to separate proteins by molecular weight.
Blots were transferred to PVDF membranes and probed with the antibodies indicated.
Membranes were imaged utilizing Licor Odyssey imager.
Each stain includes a range of molecular weight ladder of at least four markers.
Some membranes were cut to probe for multiple targets or to reduce background from RBD.
Some antibodies recognize ladders stronger than others, and intensity differences of the ladder compared to the target protein is observed.

Supplementary Figure 6

# Raw image files for Figure 3A SW837 Cells

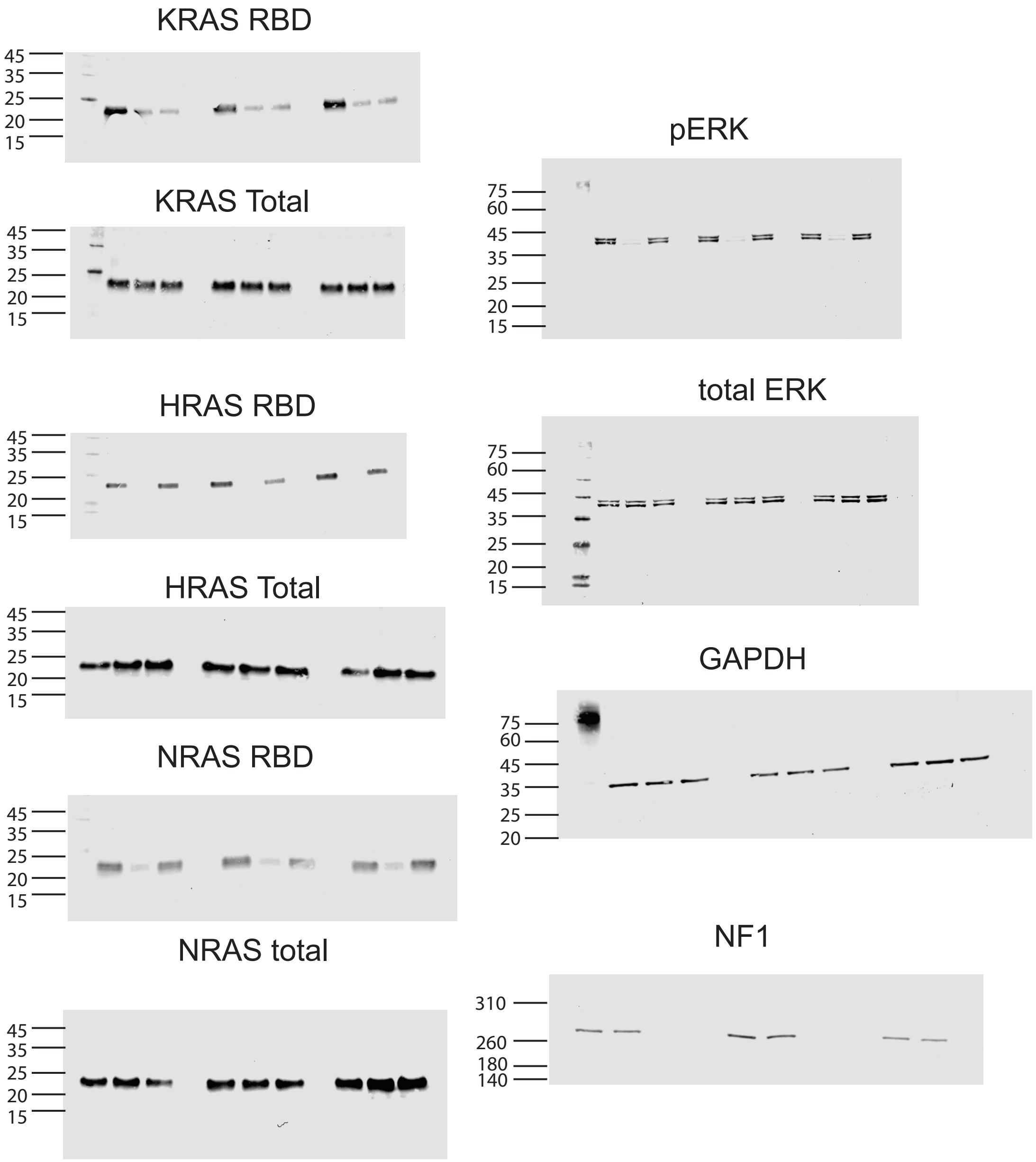


Protein lysates were run on gels to separate proteins by molecular weight.
Blots were transferred to PVDF membranes and probed with the antibodies indicated.
Membranes were imaged utilizing Licor Odyssey imager.
Each stain includes a range of molecular weight ladder of at least four markers.
Some membranes were cut to probe for multiple targets or to reduce background from RBD.
Some antibodies recognize ladders stronger than others, and intensity differences of the ladder compared to the target protein is observed.

Supplementary Figure 7

# Raw image files for Figure 3A SW1463 Cells

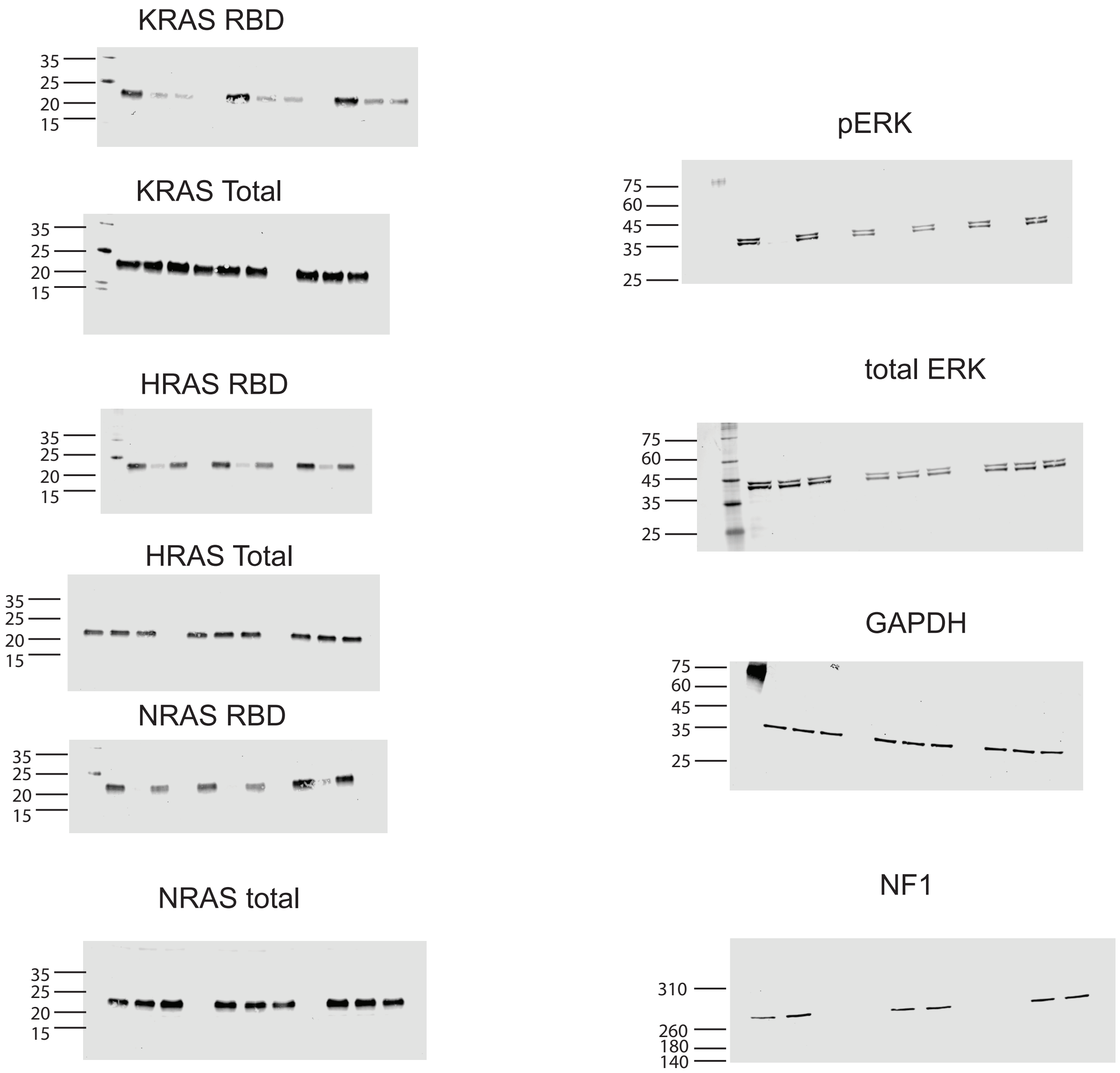


Protein lysates were run on gels to separate proteins by molecular weight.
Blots were transferred to PVDF membranes and probed with the antibodies indicated.
Membranes were imaged utilizing Licor Odyssey imager.
Each stain includes a range of molecular weight ladder of at least four markers.
Some membranes were cut to probe for multiple targets or to reduce background from RBD.
Some antibodies recognize ladders stronger than others, and intensity differences of the ladder compared to the target protein is observed.

# Supplementary Figure 8

# Raw image files for Figure 3C SW1463 Cells

KRAS RBD

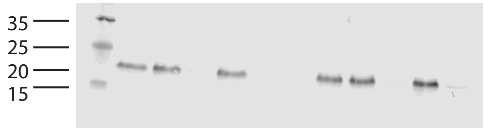


KRAS Total

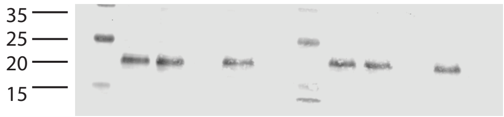


HRAS RBD

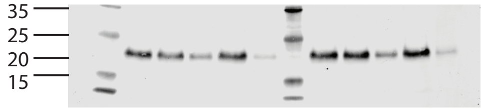


HRAS Total

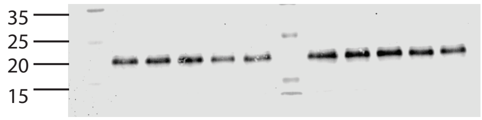


NRAS RBD

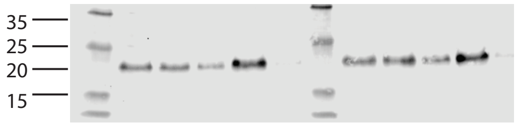


NRAS total

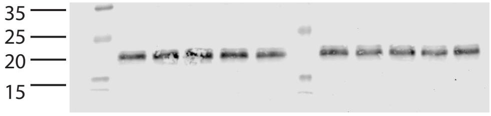


pERK

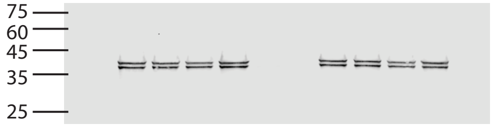


total ERK

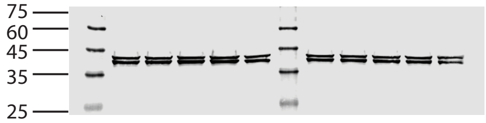


GAPDH

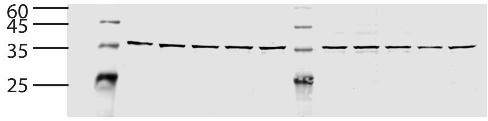


Protein lysates were run on gels to separate proteins by molecular weight.
Blots were transferred to PVDF membranes and probed with the antibodies indicated.
Membranes were imaged utilizing Licor Odyssey imager.
Each stain includes a range of molecular weight ladder of at least four markers.
Some membranes were cut to probe for multiple targets or to reduce background from RBD.
Some antibodies recognize ladders stronger than others, and intensity differences of the ladder compared to the target protein is observed.

Supplementary Figure 9

# Raw image files for Figure 3E SW1463 Cells

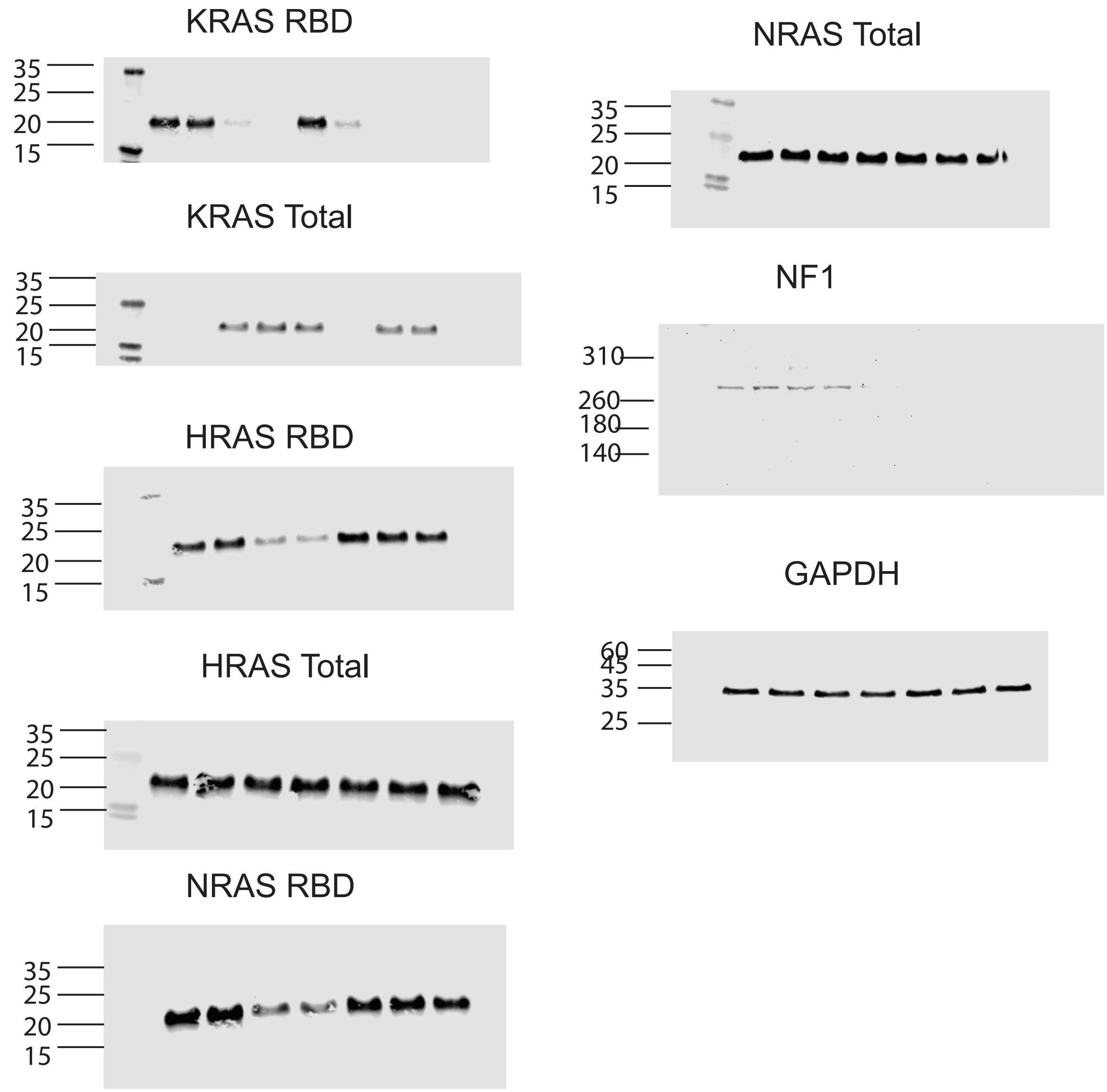


Protein lysates were run on gels to separate proteins by molecular weight.
Blots were transferred to PVDF membranes and probed with the antibodies indicated.
Membranes were imaged utilizing Licor Odyssey imager.
Each stain includes a range of molecular weight ladder of at least four markers.
Some membranes were cut to probe for multiple targets or to reduce background from RBD.
Some antibodies recognize ladders stronger than others, and intensity differences of the ladder compared to the target protein is observed.

Supplementary Figure 10
