# Supplementary material for: Co-targeting KRAS G12C and EGFR reduces both mutant and wild-type RAS-GTP
Source: NPJ Precis Oncol. 2022 Nov 23;6:86. doi: 10.1038/s41698-022-00329-w (PMC9684405; doi:10.1038/s41698-022-00329-w)
Supplement: Supplementary file 2 — REPORTING SUMMARY [file 41698_2022_329_MOESM2_ESM.pdf]

nature portfolio

Corresponding author(s): Edward Stites, Thomas McFall

Last updated by author(s): July 14, 2022

Reporting Summary

Nature Portfolio wishes to improve the reproducibility of the work that we publish. This form provides structure for consistency and transparency in reporting. For further information on Nature Portfolio policies, see our Editorial Policies and the Editorial Policy Checklist.

Statistics

For all statistical analyses, confirm that the following items are present in the figure legend, table legend, main text, or Methods section.

n/a	Confirmed	
☐	☒	The exact sample size (*n*) for each experimental group/condition, given as a discrete number and unit of measurement
☐	☒	A statement on whether measurements were taken from distinct samples or whether the same sample was measured repeatedly
☐	☒	The statistical test(s) used AND whether they are one- or two-sided<br>*Only common tests should be described solely by name; describe more complex techniques in the Methods section.*
☐	☒	A description of all covariates tested
☐	☒	A description of any assumptions or corrections, such as tests of normality and adjustment for multiple comparisons
☐	☒	A full description of the statistical parameters including central tendency (e.g. means) or other basic estimates (e.g. regression coefficient) AND variation (e.g. standard deviation) or associated estimates of uncertainty (e.g. confidence intervals)
☐	☒	For null hypothesis testing, the test statistic (e.g. *F*, *t*, *r*) with confidence intervals, effect sizes, degrees of freedom and *P* value noted<br>*Give P values as exact values whenever suitable.*
☒	☐	For Bayesian analysis, information on the choice of priors and Markov chain Monte Carlo settings
☒	☐	For hierarchical and complex designs, identification of the appropriate level for tests and full reporting of outcomes
☒	☐	Estimates of effect sizes (e.g. Cohen's *d*, Pearson's *r*), indicating how they were calculated

Our web collection on statistics for biologists contains articles on many of the points above.

Software and code

Policy information about availability of computer code

Data collection	No software was used to analyze the acquire the experimental data sets.
Data analysis	Statistical Analysis was performed by using graphpad Prism 8 software.

For manuscripts utilizing custom algorithms or software that are central to the research but not yet described in published literature, software must be made available to editors and reviewers. We strongly encourage code deposition in a community repository (e.g. GitHub). See the Nature Portfolio guidelines for submitting code & software for further information.

Data

Policy information about availability of data

All manuscripts must include a data availability statement. This statement should provide the following information, where applicable:

- Accession codes, unique identifiers, or web links for publicly available datasets
- A description of any restrictions on data availability
- For clinical datasets or third party data, please ensure that the statement adheres to our policy

The datasets generated during and/or analyzed during the current study are available from the corresponding author on reasonable request.

Human research participants

Policy information about studies involving human research participants and Sex and Gender in Research.

Reporting on sex and gender	*Use the terms sex (biological attribute) and gender (shaped by social and cultural circumstances) carefully in order to avoid confusing both terms. Indicate if findings apply to only one sex or gender; describe whether sex and gender were considered in study design whether sex and/or gender was determined based on self-reporting or assigned and methods used. Provide in the source data disaggregated sex and gender data where this information has been collected, and consent has been obtained for sharing of individual-level data; provide overall numbers in this Reporting Summary. Please state if this information has not been collected. Report sex- and gender-based analyses where performed, justify reasons for lack of sex- and gender-based analysis.*
Population characteristics	*Describe the covariate-relevant population characteristics of the human research participants (e.g. age, genotypic information, past and current diagnosis and treatment categories). If you filled out the behavioural & social sciences study design questions and have nothing to add here, write "See above."*
Recruitment	*Describe how participants were recruited. Outline any potential self-selection bias or other biases that may be present and how these are likely to impact results.*
Ethics oversight	*Identify the organization(s) that approved the study protocol.*

Note that full information on the approval of the study protocol must also be provided in the manuscript.

Field-specific reporting

Please select the one below that is the best fit for your research. If you are not sure, read the appropriate sections before making your selection.

☒ Life sciences ☐ Behavioural & social sciences ☐ Ecological, evolutionary & environmental sciences

For a reference copy of the document with all sections, see nature.com/documents/nr-reporting-summary-flat.pdf

Life sciences study design

All studies must disclose on these points even when the disclosure is negative.

Sample size	For experimental cell biology techniques, we performed at least three experiments to accurately test if findings were reproducible and statistics could determine significance.
Data exclusions	Not data was excluded in this study.
Replication	All experiments were performed three times and multiple controls were included in each experiment to ensure baseline measurements for reference. Furthermore all experiments were evaluated with the proper statistical test to ensure significance. All experiments are described in the text with number of replicates performed.
Randomization	No experimental groups (population) in this study, and all experiments were molecular mechansitic studies where randomization is not warranted.
Blinding	Investigators were not blinded during data collection.

Reporting for specific materials, systems and methods

We require information from authors about some types of materials, experimental systems and methods used in many studies. Here, indicate whether each material, system or method listed is relevant to your study. If you are not sure if a list item applies to your research, read the appropriate section before selecting a response.

Materials & experimental systems

n/a	Involved in the study
☐	☒ Antibodies
☐	☒ Eukaryotic cell lines
☒	☐ Palaeontology and archaeology
☒	☐ Animals and other organisms
☒	☐ Clinical data
☒	☐ Dual use research of concern

Methods

n/a	Involved in the study
☒	☐ ChIP-seq
☒	☐ Flow cytometry
☒	☐ MRI-based neuroimaging

Antibodies

Antibodies used

Anti ERK 1/2 Antibody, Clone W15133B, Biolegend Cat# 686902 Lot# B290205
Anti-Phosphorylated ERK 1/2 Clone 4B11B69, Biolegend, Cat#675502, Lot# B292934- company confirmed by loss/gain of expression
anit-GAPDH Clone FF26A/F9, Biolegend, Cat# 649202, Lot#B299509- compnay confromed by loss/gain of function
Anti-HRAS, Abcam, Cat#32417, Lot#GR244237-2-company validated by loss/gain preseason-investiagtor loss of expression (siRNA)
anti-KRaS Sigma, CAT#H00003845-M01 Lot#M3141-S2-company validated by loss/gain preseason-investiagtor loss of expression (siRNA)
Anti-NRAS, Abcam, ab167136 Lot#GR3327003-18, company validated by loss/gain expression-investiagtor loss of expression (siRNA)
Anti-NF1, Santa Cruz, CAT# SC-20017 LoT# c0713, company validated by loss/gain expression-investiagtor loss of expression (siRNA)

Validation

All antibodies were validate by the manufacturers by gain and loss of expression techniques and were raised against purified protein. We have also validate by siRNA experiments.

Eukaryotic cell lines

Policy information about cell lines and Sex and Gender in Research

Cell line source(s)

SW48 Isogenics were supplied by Horizon Discovery, all other cell lines in this study were supplied from ATCC.

Authentication

All cell lines were purchased new for this study, and were authenticated by sequencing before being shipped to investigators.

Mycoplasma contamination

All cells were confirmed to be mycoplasm free on arrival in the lab, cells are routinely tested every six months by light scattering media test.

Commonly misidentified lines
(See ICLAC register)

NA
